# Elastoplastic analysis on deformation and failure characteristics of surrounding rock of soft-coal roadway based on true triaxial loading and unloading tests

**DOI:** 10.1038/s41598-024-72052-4

**Published:** 2024-09-10

**Authors:** Chongyang Jiang, Lianguo Wang, Jiaxing Guo, Shuai Wang

**Affiliations:** State Key Laboratory of Intelligent Construction and Healthy Operation and Maintenance of Deep Underground Engineering, China University of Mining and Technology, Xuzhou, 221116 China

**Keywords:** True triaxial loading and unloading, Deformation and strength characteristics of soft coal, Mogi–Coulomb criterion, Nonlinear hardening and softening model, Elastoplastic analysis on roadway, Petrology, Civil engineering

## Abstract

Since accidents such as roof caving, rock fragmentation, and severe deformation are particularly likely to occur during roadway excavation in soft and thick coal seams, grasping the range and distribution of deformation and fracturing of surrounding rock is of crucial for evaluating roadway stability and optimizing support design in such coal seams. In this study, based on the stress paths encountered during roadway excavation, true triaxial loading and unloading tests were carried out on soft coal, and the deformation and strength evolutions of soft coal under different intermediate principal stress conditions were analyzed. The test results show that the stress–strain relationship in the pre-peak plasticity-strengthening and post-peak plasticity-weakening stages follows a quadratic function, and the strengeth evolution conforms to the Mogi–Coulomb criterion. Moreover, analytical solutions for the displacement of surrounding rock, the radius of the broken zone, and the radius of the plastic zone of soft-coal roadways under excavation stress paths were derived after taking the nonlinear hardening and softening characteristics of the strain of soft coal, the Mogi–Coulomb criterion, the intermediate principal stress, and the dilatancy characteristics of surrounding rock into comprehensive consideration. Finally, in accordance with a practical engineering case, the influences of the intermediate principal stress coefficient, the lateral pressure coefficient, and the support force on the deformation and failure characteristics of the soft-coal roadway were analyzed. The analysis reveals that an increase in intermediate principal stress aggravates the deformation of surrounding rock and enlarges the plastic and broken zones; variations in the lateral pressure coefficient alter the shape of the broken zone and the distribution of surface displacement; and an increase in the support force effectively reduces the plastic zone, broken zone, and surface displacement of the roadway. The research results can provide valuable theoretical basis for the stability evaluation and support design of soft-coal roadways.

## Introduction

With the depletion of shallow coal resources, deep mining has become the norm in the coal industry^1,2^. Affected by high ground stress in the deep, coal seams tend to become soft and fractured. During the excavation of a roadway in such soft and thick coal seams, large broken and plastic zones will emerge in the surrounding rock, posing safety hazards such as roof caving, rock fragmentation, and severe deformation^3^. Given the above fact, grasping the range and distribution of deformation, broken zone, and plastic zone in the surrounding rock is crucial for evaluating roadway stability and optimizing support design in such coal seams^4^. However, different constitutive models and strength criteria can lead to significant differences in the theoretical calculation results of the deformation and failure characteristics of the surrounding rock. Therefore, aming at accurately calculating the deformation and failure characteristics of the surrounding rock, it is meaningful to select a constitutive model and a strength criterion that are consistent with actual engineering.

Roadway excavation is actually a loading and unloading process for coal in a true triaxial stress state^5,6^. In this process, the stress of the surrounding rock of a roadway gradually transitions from being triaxial to being biaxial or unidirectional^7,8^, and the surrounding rock undergoes changes of vertical stress loading and horizontal stress unloading^9^. Numerous scholars have conducted plentiful loading and unloading tests on coal and rock according to the excavation and unloading process. Feng et al.^10–12^ carried out loading and unloading tests on hard rock (e.g., marble) under different stress paths and explored the strength, deformation, and failure mechanisms of hard rock under these paths. Li et al.^13^ conducted true triaxial loading and unloading tests on sandstone with different intermediate principal stresses and revealed the influences of the intermediate principal stress and the unloading action on sandstone dilatancy. Through true triaxial loading and unloading tests on columnar jointed rock specimens with different inclinations, Que et al.^14^ uncovered the impact of excavation and unloading on the anisotropy strength and energy evolution of columnar jointed rock. Liu et al.^15^ studied the changes in acoustic emission parameters of red sandstone during its true triaxial unloading failure and established the relationship between the intermediate principal stress and the acoustic emission information. Wang et al.^16^ performed unloading tests on coal samples with the aid of a true triaxial fluid–structure interaction testing system, and obtained their deformation, damage, and failure characteristics. Liang et al.^17^ conducted true triaxial loading and unloading tests on coal under different stress paths and analyzed the changes in deformation parameters, energy distribution, and fracture characteristics of coal under these paths. Currently, experimental research on true triaxial loading and unloading is mainly carried out on marble, granite, sandstone, hard coal, etc., while that on soft and fractured coal is scarcely reported.

In terms of elastoplastic analysis, relavent scholars have conducted extensive research on the deformation and failure characteristics of surrounding rock of roadways using different constitutive models and yield criteria of coal and rock. Sharan^18^, Sofianos and Nomikos^19^, Lee and Pietruszczak^20^, and Lv et al.^21^ considered different constitutive models of rock based on the Hoek–Brown strength criterion and the non-correlated flow rule, and made elastoplastic analysis on the surrounding rock of roadways. Moreover, they derived analytical solutions for the deformation and failure range of surrounding rock. Zareifard and Fahimifar^22^, Park^23^, and Ranjbarnia et al.^24^ derived analytical solutions for the stress and displacement of surrounding rock under hydrostatic pressure fields based on the linear strain softening model and the Mohr–Coulomb criterion. They also analyzed the influence of softening parameters on the stress and displacement of surrounding rock through numerical examples. Ghorbani and Hasanzadehshooiili^25^, Zhang et al.^26^, Yuan et al.^27^, and Wang et al.^28^ integrated the intermediate principal stress, strain softening characteristics, rock shear dilation parameters, and Young’s modulus variations. Based on the Drucker–Prager criterion or the unified strength theory, they derived analytical solutions for the stress, displacement, and plastic zone of surrounding rock of circular roadways. Based on the Mohr–Coulomb and Drucker–Prager strength criteria, Jing et al.^29^ derived the elastoplastic solution for the surrounding rock by combining rock rheology and long-term strength tests, and verified it with engineering examples. Overall, scholars have conducted abundant research on the analytical solutions for deformation and failure of roadways. However, most of the existing elastoplastic solutions for roadways are theoretically derived based on the constitutive relationship and strength criteria of coal and rock masses under compressive loading conditions, which differ from the stress changes encountered during roadway excavation and have certain limitations. The above research status nessesiates performing true triaxial loading and unloading tests based on the stress change characteristics of surrounding rock during the excavation of soft-coal roadways and establishing corresponding elastoplastic analysis models.

In this study, the evolutions of deformation and strength characteristics of soft coal under true triaxial loading and unloading conditions were investigated, and the constitutive relationship and strength criterion of soft coal under real stress paths during roadway excavation were determined. On this basis, the analytical solutions for the displacement of surrounding rock, the radius of the broken zone, and the radius of the plastic zone of soft-coal roadways under excavation stress paths were derived by taking the intermediate principal stress and the dilatancy characteristics of surrounding rock into account. Finally, in accordance with a practical engineering case, the effects of different influencing factors on the deformation and failure characteristics of the soft-coal roadway were analyzed. The research results can provide essential theoretical basis for the stability evaluation and support design of soft-coal roadways.

## True triaxial loading and unloading tests on soft coal

### Sample preparation

**Fig. 1:**
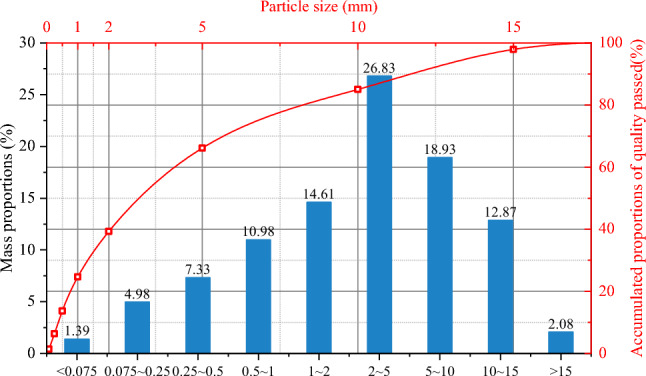
Size distribution of raw coal particle.

The coal samples used in this experiment were collected from #8 coal seam in Huaibei Mining Area, China. This coal seam is of an extremely low strength and basically appears in a fragmented and loose state. Since such soft and fractured coal is difficult to be sampled and directly used for tests, it was processed into equivalent soft coal samples after being collected on site. The loose raw coal has a moisture content of approximately 3.25%. Prior to sample preparation, the raw coal particles were screened using sieves with pore sizes of 0.075 mm, 0.25 mm, 0.5 mm, 1 mm, 2 mm, 5 mm, 10 mm, and 15 mm, respectivly, based on which the raw coal samples were then classified. The mass proportions and particle size distribution curves for the raw coal were calculated (Fig. 1). As illustarted in Fig. 1, the particle size of the raw coal predominantly ranges from 0 to 15 mm. Particles within this size range account for 97.92% of the total mass, while those larger than 15 mm, mostly consisting of small gangue, account for only 2.08% and are considered unrepresentative. Therefore, raw coal particles within the size range of 0–15 mm were selected for testing, and samples were prepared based on their proportional distribution. This approach not only replicates the grading composition of the raw coal but also avoids test errors arising from uneven packing density and particle arrangement, thereby ensuring sample uniformity.

**Fig. 2:**
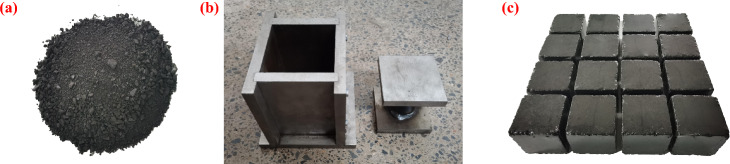
Sample mold and soft coal samples: (**a**) raw coal: (**b**) sample mold; (**c**) part of coal samples.

Considering that the actual buried depth of the coal seam was 850 m, the forming pressure of the coal samples was set to 22 MPa. Following several preliminary tests, it was determined that 1480 g of loose raw coal was required for preparing a coal sample. The coal particles were weighed according to their respective size proportions, uniformly mixed (Fig. 2a), and then added to the briquette pressing mold shown in Fig. 2b, where it was subjected to slowly increasing loads through a testing machine until the forming pressure was reached. Afterwards, the pressure was maintained for 20 min before the cubic soft coal sample with a size of 100 mm × 100 mm × 100 mm was taken out (Fig. 2c). Finally, the obtained samples were wrapped with cling films for later use. To ensure homogeneity of the samples, wave velocity tests were carried out to remove highly dispersed ones. The test results show that the wave velocities of the selected samples range from 0.279 to 0.364 km/s, with an average of 0.331 km/s.

### Experimental equipment

**Fig. 3:**
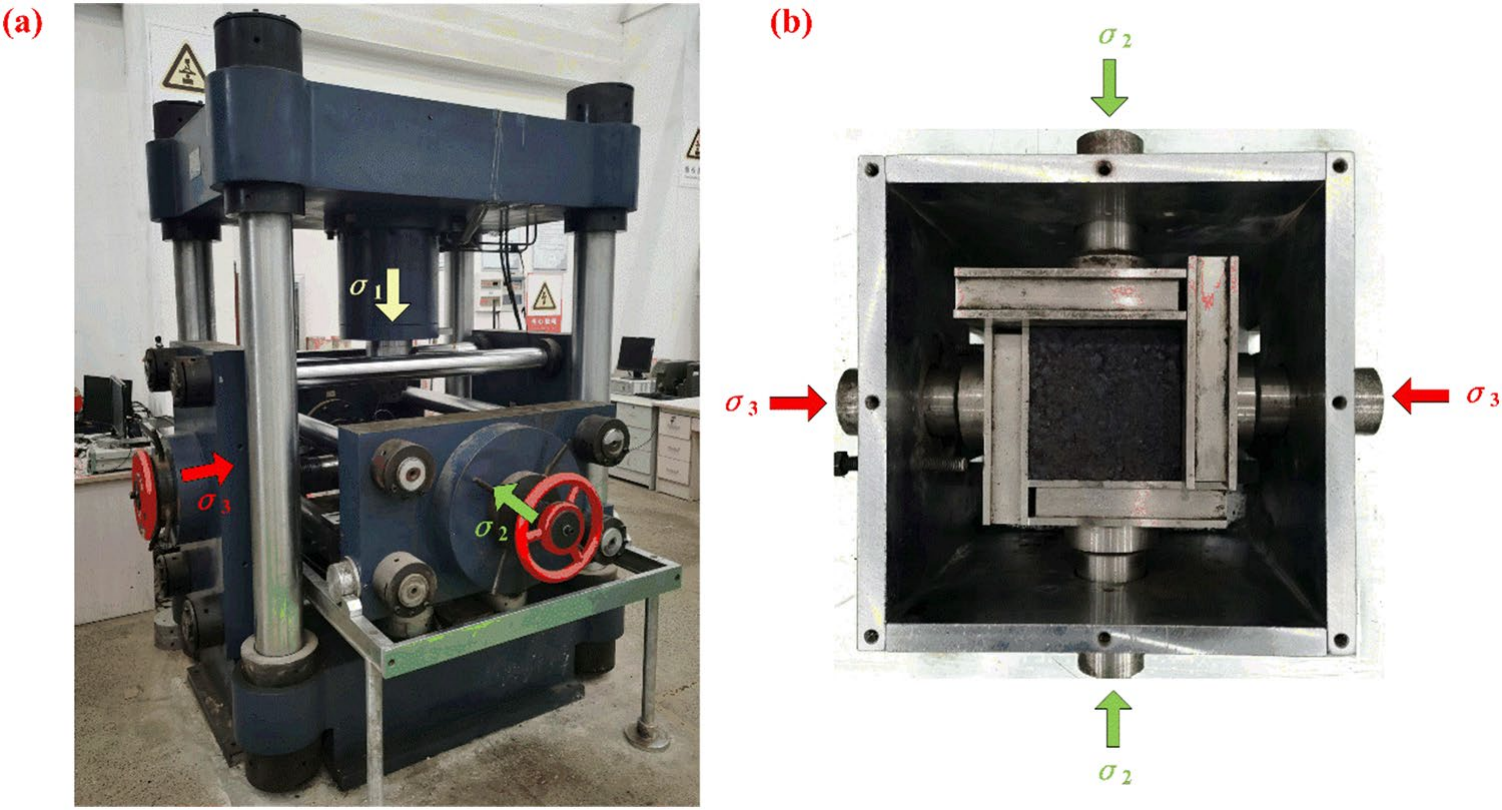
True triaxial electro-hydraulic servo loading test system: (**a**) true triaxial testing machine; (**b**) true triaxial pressure chamber.

The true triaxial loading and unloading tests were carried out with the aid of a true triaxial electro-hydraulic servo loading test system from China University of Mining and Technology. This system mainly consists of a three-way servo control loading system, an automatic acquisition system, a true triaxial pressure chamber, and an acoustic emission monitoring system^30^. As displayed in Fig. 3a, the three-way servo control loading system comprises three mutually perpendicular and independent loading subsystems $\sigma_{1}$, $\sigma_{2}$, and $\sigma_{3}$, which can achieve independent servo loading control and simulate the real stress state of rock masses in underground engineering. The maximum servo loading pressures in the three directions are 1,600 kN, 500 kN, and 300 kN, respectively. The measurement accuracies of the test system for force and deformation are 0.01 kN and 0.002 mm, respectively. As shown in Fig. 3b, the true triaxial pressure chamber, located at the center of the three-way servo control loading system, is composed of a pressure box, a base, and loading plates. The sample and the loading plates are interlocked, which allows for loading on the sample in three main stress directions.

### Experimental scheme

**Fig. 4:**
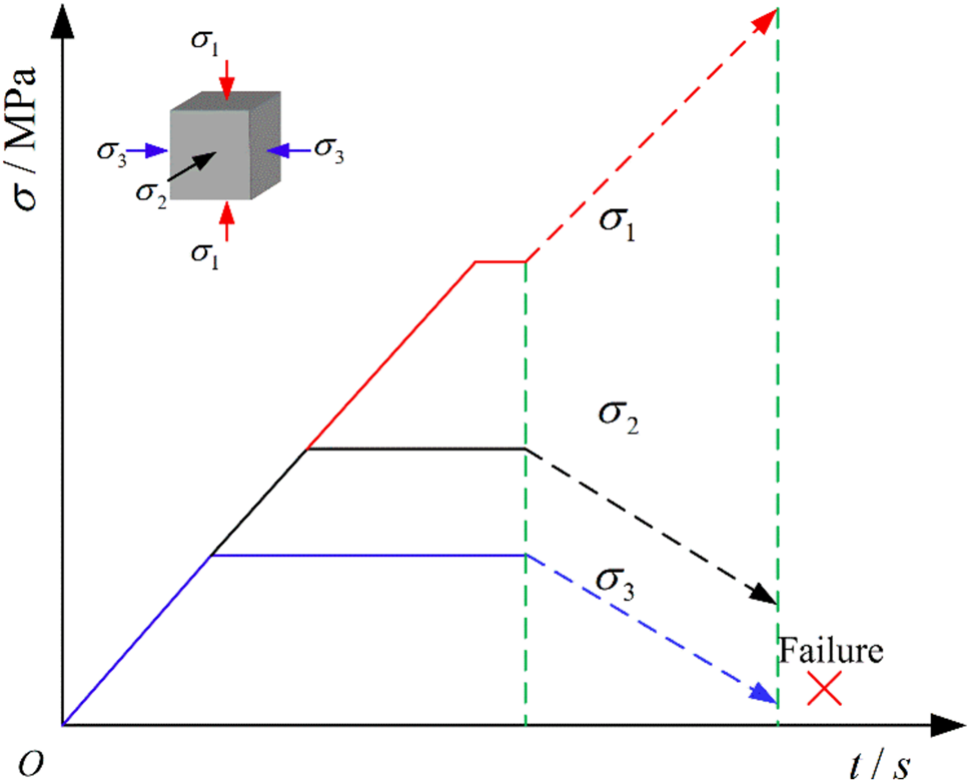
Schematic diagram of the stress path in the true triaxial loading and unloading tests.

According to field measurements, the maximum principal stress ($\sigma_{1}$) is approximately 22 MPa, the intermediate principal stress ($\sigma_{2}$) ranges from 13.57 MPa to 21 MPa, and the minimum principal stress ($\sigma_{3}$) is around 10 MPa. Before roadway excavation, the surrounding rock is in a three-dimensional stress equilibrium state. During excavation, the stress state of the surrounding rock changes. To be specific, stress concentration and stress unloading occur, typically characterized by an increase in $\sigma_{1}$ and a gradual decrease in $\sigma_{2}$ and $\sigma_{3}$. Affected by stress changes, soft coal fractures and becomes unstable, which results in deformation and failure of the surrounding rock. In addition, the intermediate principal stress significantly influences the deformation and failure of the surrounding rock. Therefore, in the hope of exploring the deformation and failure characteristics of soft coal in the presence of excavation-dincued disturbance, five sets of true triaxial loading and unloading tests under different intermediate principal stress conditions were designed in this experiment. The test scheme is disclosed in Table 1, and the specific stress path is illustrated in Fig. 4. Considering the discreteness of the experimental results, each group of tests was repeated three times.

**Table 1:** True triaxial loading and unloading test scheme on soft coal.

Test type	Initial stress /MPa			Stress path	No.
	$\sigma_{1}$	$\sigma_{2}$	$\sigma_{3}$		
True triaxial loading and unloading	22	14	10	Loading $\sigma_{1}$ and unloading $\sigma_{2}$, $\sigma_{3}$ simultaneously	L1-U23-1
	22	16	10		L1-U23-2
	22	18	10		L1-U23-3
	22	20	10		L1-U23-4
	22	22	10		L1-U23-5

As illustrated in Fig. 4, a tested coal sample was first loaded to the preset initial true triaxial stress state. Then, $\sigma_{1}$, $\sigma_{2}$, and $\sigma_{3}$ were synchronously applied to the sample at a rate of 0.2 MPa/s until the target hydrostatic pressure state (10 MPa) was reached. Subsequently, $\sigma_{3}$ remained unchanged, and $\sigma_{2}$ was loaded to the preset value at a rate of 0.2 MPa/s. After that, $\sigma_{2}$ and $\sigma_{3}$ remained constant, and $\sigma_{1}$ was loaded to the preset value at a rate of 0.2 MPa/s and then remained unchanged. At this time, the initial true triaxial stress state was reached. Based on relevant studies^31,32^ and multiple pre-experiments, it was concluded that a lower loading and unloading rate conduces to effectively preventing sudden failure or instability of the sample, allowing for a more accurate capture of the stress–strain relationship in soft coal. Acorddingly, $\sigma_{1}$ was loaded at a rate of 0.1 MPa/s, while $\sigma_{2}$ and $\sigma_{3}$ were simultaneously unloaded at a rate of 0.1 MPa/s until the specimen failed.

### Analysis and discussion of test results

#### Deformation characteristics of soft coal

**Fig. 5:**
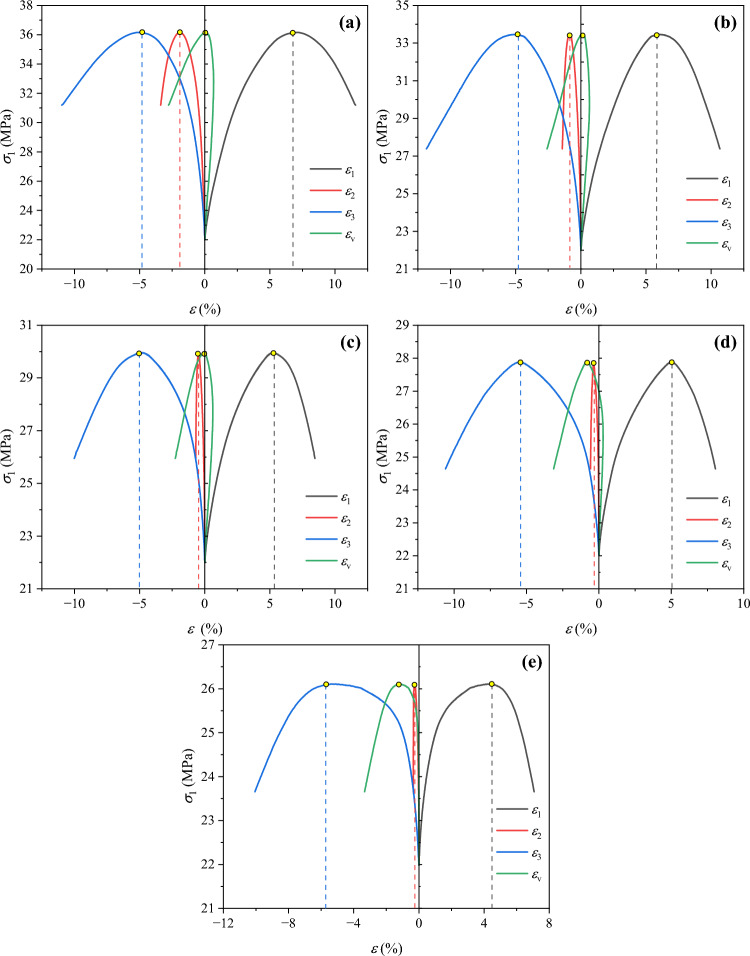
Stress–strain curves of soft coal under true triaxial loading and unloading conditions: (**a**) $\sigma_{2}$ = 14 MPa; (**b**) $\sigma_{2}$ = 16 MPa; (**c**) $\sigma_{2}$ = 18 MPa; (**d**) $\sigma_{2}$ = 20 MPa; (**e**) $\sigma_{2}$ = 22 MPa.

**Fig. 6:**
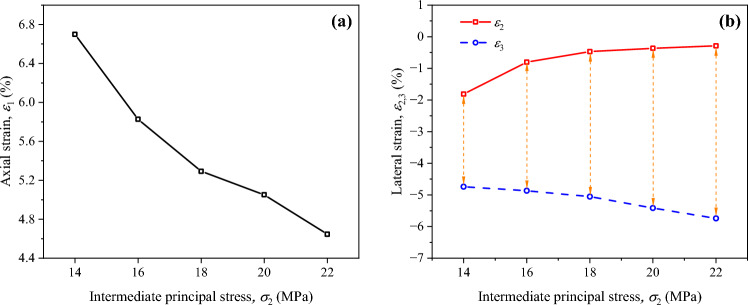
Variation curves of peak strains of soft coal under different intermediate principal stress conditions: (**a**) axial strain *ε*_1_; (**b**) lateral strains *ε*_2_ and *ε*_3._

To facilitate comparison, the deformation of the samples in the initial stress state after loading was taken as the starting point, and only the data of subsequent loading and unloading processes were analyzed. Figure 5 presents the variation curves of axial stress $\sigma_{1}$ of soft coal with axial strain $\varepsilon_{1}$, lateral strains $\varepsilon_{2}$ and $\varepsilon_{3}$, and volumetric strain $\varepsilon_{v}$ under true triaxial loading and unloading conditions, and Fig. 6 displays the variation curves of peak strains with the intermediate principal stress when the peak stress of soft coal is reached.

It can be observed from Fig. 5 that the stress–strain curves of soft coal under these five intermediate principal stress conditions can be divided into four stages, namely the elastic stage (Stage I), the pre-peak plasticity-strengthening (hardening) stage (Stage II), the post-peak plasticity-weakening (softening) stage (Stage III), and the instability failure stage (Stage IV). In Stage I, the stress of coal changes roughly linearly with the strain. As the loading and unloading test proceeds, the coal enters Stage II where the axial stress $\sigma_{1}$ rises nonlinearly at a gradually decelerated rate. After reaching the peak, it starts to decline progressively. In Stage III, the axial stress $\sigma_{1}$ decreases nonlinearly, while the strains in the $\sigma_{2}$ and $\sigma_{3}$ unloading directions surge. The dilatancy and deformation in the $\sigma_{3}$ direction are more notable than those in the $\sigma_{2}$ direction. Eventually, the coal loses its bearing capacity in Stage IV. In Stages II and III, the stress–strain relationship basically follows a quadratic function, whose maximum value is exactly the peak stress of coal. Besides, the volumetric strain tends to increase first and then decrease throughout the loading and unloading process, indicating that the deformation pattern of coal transitions from axial compression in the initial stage to lateral dilatancy later stage.

It can be found from Fig. 6 that at the initial $\sigma_{2}$ of 14 MPa, the values of $\sigma_{2}$ and $\sigma_{3}$ differ slightly, so do the values of $\varepsilon_{2}$ and $\varepsilon_{3}$ when the peak strength is reached. At this time, the lateral deformation of coal remains coordinated, and its bearing capacity is relatively strong. As the initial $\sigma_{2}$ increases, the difference between values of $\sigma_{2}$ and $\sigma_{3}$ enlarges gradually. When the peak strength coal is reached, $\varepsilon_{1}$ gradually decreases; $\varepsilon_{2}$ gradually declines and tends to level off; and $\varepsilon_{3}$ progressively rises. That is, the difference between $\varepsilon_{2}$ and $\varepsilon_{3}$ enlarges correspondingly. These results indicate that an increase in $\sigma_{2}$ can accelerate the failure of coal, which makes the coal more prone to dilatancy and deformation in the $\sigma_{3}$ direction.

#### Discussion on the applicability of strength criteria

**Fig. 7:**
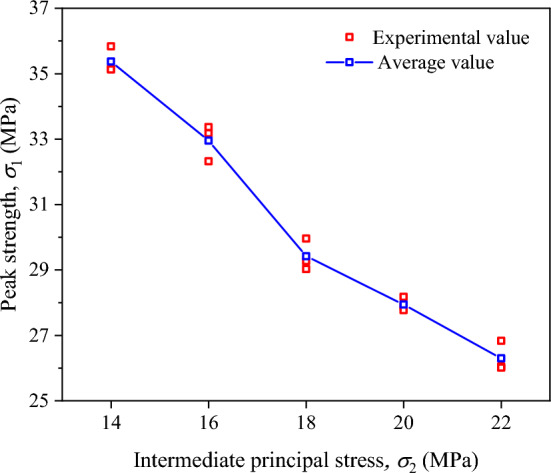
Variations of the peak strength of coal under true triaxial loading and unloading conditions.

The variation of the peak strength of coal with the intermediate principal stress under true triaxial loading and unloading conditions is exhibited in Fig. 7. Clearly, the peak strength gradually decreases with the increase in initial $\sigma_{2}$. Specifically, it decreases from 35.37 MPa at the $\sigma_{2}$ of 14 MPa to 26.3 MPa at the $\sigma_{2}$ of 22 MPa, a decrease of 25.64%. The reason for this phenomenon is that an increase in $\sigma_{2}$ restrains dilatancy in the $\sigma_{2}$ direction and promotes dilatancy in the $\sigma_{3}$ direction, thereby accelerating the failure of coal.

The Mohr–Coulomb criterion, Drucker–Prager criterion, and Mogi–Coulomb criterion, three common criteria for coal strength, were selected for investigating the applicability of different strength criteria in true triaxial loading and unloading tests on soft coal.

According to the Mohr–Coulomb criterion, the principal stress can be written as:

1 $$ \sigma_{1} = \frac{1 + \sin \phi }{{1 - \sin \phi }}\sigma_{3} + \frac{2c\cos \phi }{{1 - \sin \phi }} $$

where $\phi$ is the friction angle of coal; and *c* is the cohesion.

Based on the Drucker–Prager criterion, the principal stress can be expressed by:

2 $$ \sqrt {J_{2} } = \alpha I_{1} + K $$

3 $$ I_{1} = \sigma_{1} + \sigma_{2} + \sigma_{3} $$

4 $$ J_{2} = \frac{1}{6}\left[ {(\sigma_{1} - \sigma_{2} )^{2} + (\sigma_{1} - \sigma_{3} )^{2} + (\sigma_{2} - \sigma_{3} )^{2} } \right] $$

where $I_{1}$ is the first invariant of stress; $J_{2}$ is the second invariant of stress bias; and $\alpha$ and $K$ are experimental constants related to the friction angle $\phi$ and the cohesive force *c*, which can be calculated as:

5 $$ \left\{ \begin{gathered} \alpha = \frac{2\sin \phi }{{\sqrt 3 (3 - \sin \phi )}} \hfill \\ K = \frac{6c\cos \phi }{{\sqrt 3 (3 - \sin \phi )}} \hfill \\ \end{gathered} \right. $$

The Mogi–Coulomb criterion is an empirical criterion based on plentiful true triaxial test results^33^. According to this criterion, a specimen has yielded or failed if the octahedral shear stress $\tau_{{{\text{oct}}}}$ on any side of it reaches the limit value, as expressed by:

6 $$ \tau_{{{\text{oct}}}} = a_{1} + a_{2} \sigma_{m,2} $$

7 $$ \tau_{{{\text{oct}}}} = \frac{1}{3}\sqrt {(\sigma_{1} - \sigma_{2} )^{2} + (\sigma_{1} - \sigma_{3} )^{2} + (\sigma_{2} - \sigma_{3} )^{2} } $$

8 $$ \sigma_{m,2} = \frac{{\sigma_{1} + \sigma_{3} }}{2} $$

where $\tau_{{{\text{oct}}}}$ is the octahedral shear stress; $\sigma_{m,2}$ is the average stress; and $a_{1}$ and $a_{2}$ are experimental constants related to the friction angle $\phi$ and the cohesion *c*, which can be calculated as:

9 $$ \left\{ \begin{gathered} a_{1} = \frac{2\sqrt 2 }{3}c_{i} \cos \phi_{i} \hfill \\ a_{2} = \frac{2\sqrt 2 }{3}\sin \phi_{i} \hfill \\ \end{gathered} \right. $$

**Fig. 8:**
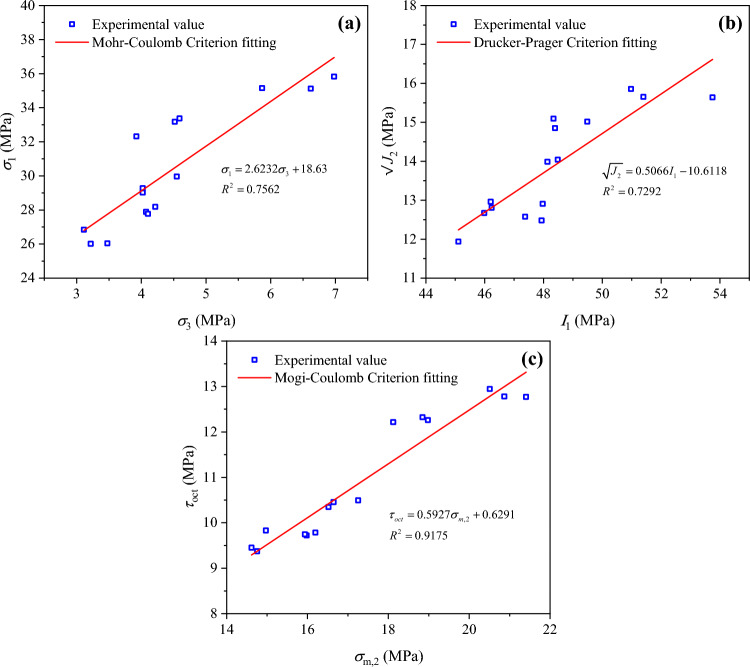
Fitting results based on the three strength criteria: (**a**) Mohr–Coulomb criterion; (**b**) Drucker–Prager criterion; (**c**) Mogi–Coulomb criterion.

The strength of soft coal under the true triaxial loading and unloading path was fitted based on the above three strength criteria, and the fitting results are given in Fig. 8.

As can be seen from the fitting results (Fig. 8), the coefficient of determination (*R*^2^) of the Mohr–Coulomb criterion is 0.76, which represents a low fitting degree. As this criterion does not consider the influence of the intermediate principal stress, it fails to accurately reflect the strength characteristics of soft coal under the true triaxial loading and unloading path. Meanwhile, the *R*^2^ of the Drucker–Prager criterion is only 0.73, which represents a low fitting degree, so this criterion is also not suitable for describing the strength characteristics of soft coal under loading and unloading conditions. In contrast, the *R*^2^ of the Mogi–Coulomb criterion reaches 0.92, demonstrating its excellent fitting effect. Hence, the Mogi–Coulomb criterion is the most effective in describing the strength characteristics of soft coal under the true triaxial loading and unloading path. Finally, the fitting parameters were utilized to calculate the cohesion and internal friction angle of soft coal, which turned to be 0.858 MPa and 38.95°, respectively.

## Elastoplastic analysis on surrounding rock of soft-coal roadway during its deformation and failure

### Mechanical model of soft-coal roadway

**Fig. 9:**
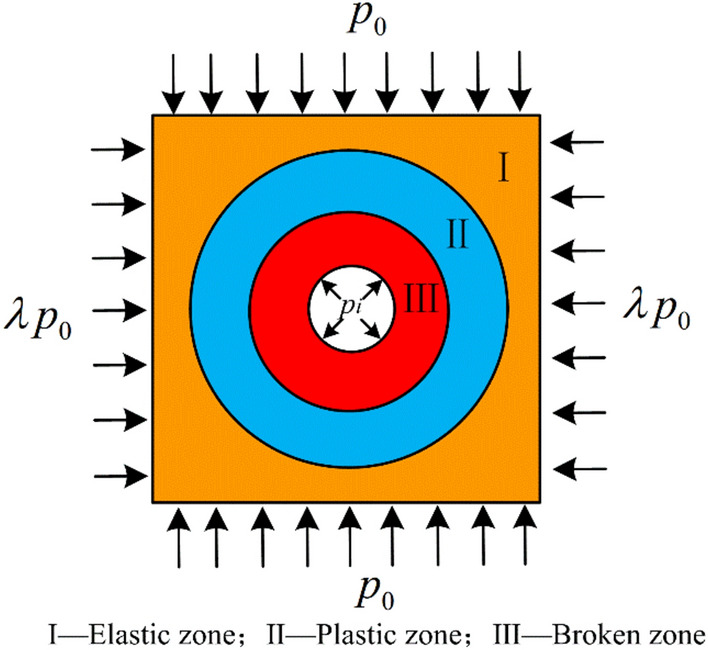
Mechanical model of the roadway.

The geological conditions of roadways are complex in practical engineering. To facilitate the theoretical elastoplastic analysis on soft-coal roadways, the “equal-area method” was adopted when constructing the mechanical model, and the circular arch-shaped roadway was equivalent to a circle with a radius of $R_{0}$. As displayed in Fig. 9, the surrounding rock of the roadway is divided into a broken zone, a plastic zone, and an elastic zone. In practical engineering, the roadway excavation direction is typically parallel to the horizontal principal stress direction in order to minimize roadway deformation and damage. In view of this fact, this paper assumes that the three principal stress directions are either orthogonal or parallel to the roadway axis. The vertical and horizontal stresses applied to the roadway model are *p*_0_ and *λp*_0_, respectively; $\lambda$ is the lateral pressure coefficient; and $p_{i}$ is the support force of the roadway.

**Fig. 10:**
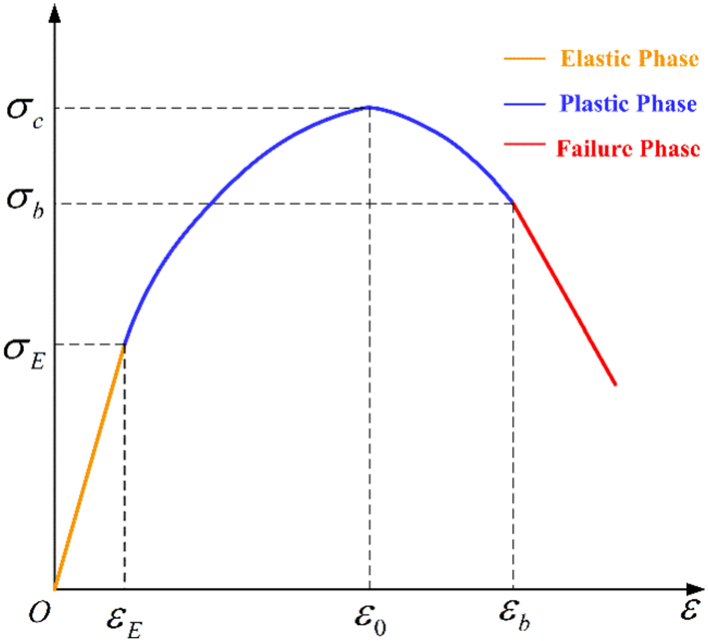
Stress–strain relationships in different stages.

It has been uncovered from the stress–strain curves of soft coal in the true triaxial loading and unloading tests that coal stays in an elastic state in the early stage of loading and unloading. When the limit elastic strength is reached, the coal enters the plastic stage, and its strain strengthens nonlinearly. After the peak stress is reached, the strain tends to weaken nonlinearly. Finally, the coal enters the failure stage. As depicted in Fig. 10, the stress is linearly correlated with the strain in both the elastic stage and the failure stage, while their correlation follows a quadratic function in the plastic phase (i.e., the pre-peak hardening and post-peak softening stages). In Fig. 10, $\varepsilon_{E}$ is the ultimate elastic strain of coal; $\varepsilon_{0}$ is the peak strain of coal; $\varepsilon_{b}$ is the failure strain of coal; $\sigma_{E}$ is the ultimate elastic stress of coal; $\sigma_{c}$ is the peak stress of coal; and $\sigma_{b}$ is the failure stress of coal.

It is assumed that the stress $\sigma_{p}$ and the strain $\varepsilon_{p}$ of coal in the plastic phase (both hardening and softening stages) satisfy the quadratic function:

10 $$ \sigma_{p} = D_{1} (\varepsilon_{p} - \varepsilon_{0} )^{2} + D_{2} (\varepsilon_{p} - \varepsilon_{0} ) + D_{3} $$

where *D*_1_, *D*_2_, and *D*_3_ are constants.

At the peak strain $(\varepsilon_{0} ,\sigma_{c} )$ in Fig. 10,

11 $$ \sigma_{c} = D_{3} $$

12 $$ \frac{{d\sigma_{p} }}{{d\varepsilon_{p} }}\left| {_{{\varepsilon_{p} = \varepsilon_{0} }} } \right. = 2D_{1} (\varepsilon_{p} - \varepsilon_{0} ) + D_{2} = 0 $$

In Fig. 10, the elastic and plastic curves are smoothly connected and have the same slope *E*. At the ultimate elastic strain $(\varepsilon_{E} ,\sigma_{E} )$, the stress and strain in the plastic stage follow the quadratic relationship:

13 $$ \frac{{d\sigma_{p} }}{{d\varepsilon_{p} }}\left| {_{{\varepsilon_{p} = \varepsilon_{e} }} } \right. = 2D_{1} (\varepsilon_{p} - \varepsilon_{0} ) + D_{2} = E $$

By combining Eqs. (11–13), this quadratic relationship can be rewritten as:

14 $$ \sigma_{p} = \frac{E}{{2(\varepsilon_{E} - \varepsilon_{0} )}}(\varepsilon_{p} - \varepsilon_{0} )^{2} + \sigma_{c} $$

It has been verified in the above section that the Mogi–Coulomb criterion can effectively describe the strength characteristics of soft coal under true triaxial loading and unloading conditions. Thus, this criterion is employed for identifying surrounding rock failure in theoretical calculation here. In practical engineering, the intermediate principal stress coefficient *b* is often introduced to denote the relationship between the three principal stresses, which is defined as:

15 $$ b = \frac{{\sigma_{2} - \sigma_{3} }}{{\sigma_{1} - \sigma_{3} }} $$

The elastoplastic problem of the surrounding rock can be solved as a plane strain problem. Then, the stress state of the surrounding rock follows:

16 $$ \sigma_{1} = \sigma_{\theta } ,\sigma_{2} = \sigma_{z} ,\sigma_{3} = \sigma_{r} $$

where $\sigma_{\theta }$, $\sigma_{z}$, and $\sigma_{r}$ are the tangential stress, axial stress, and radial stress of the surrounding rock, respectively.

By combining Eqs. (6), (7), (8), (15), and (16), the Mogi–Coulomb criterion can be expressed as:

17 $$ \sigma_{\theta i} = M_{i} \sigma_{ri} + N_{i} $$

where $M_{i} = \frac{{3a_{2} + 2\sqrt {2b^{2} - 2b + 2} }}{{2\sqrt {2b^{2} - 2b + 2} - 3a_{2} }}$ ; $N_{i} = \frac{{6a_{1} }}{{2\sqrt {2b^{2} - 2b + 2} - 3a_{2} }}$; the subscript *i* can be replaced by “*p*” and “*b*”, which represent the plastic zone and the broken zone of the surrounding rock, respectively.

**Fig. 11:**
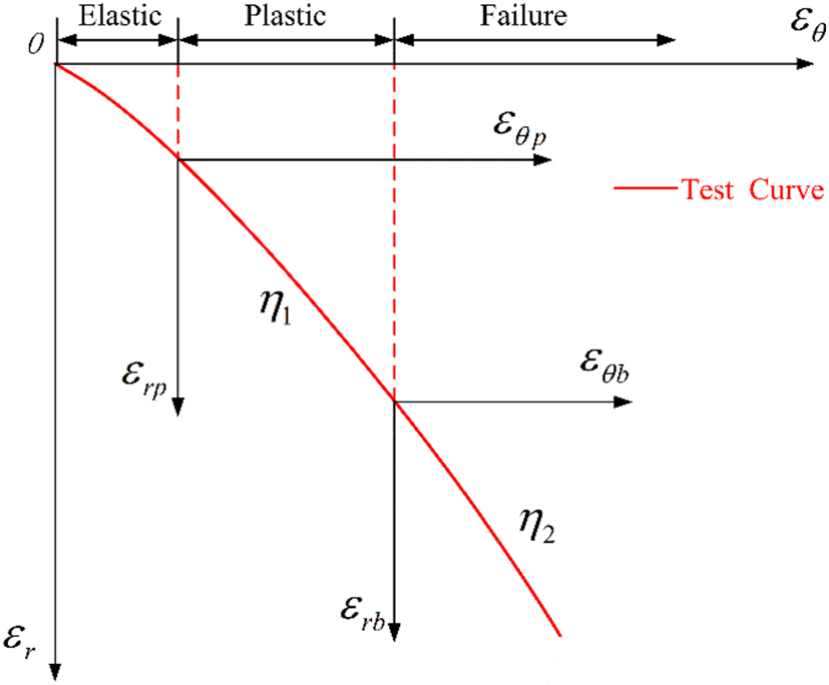
Dilatancy model of surrounding rock.

The surrounding rock will fracture and dilate when it fails. Based on the experimental results, the relationship between the dilatancy coefficient and the strain was plotted (Fig. 11).

In Fig. 11, the tangential and radial strains in the plastic and broken zones of the surrounding rock satisfy the non-correlated flow rule:

18 $$ \varepsilon_{rp} + \eta_{1} \varepsilon_{\theta p} = 0 $$

19 $$ \varepsilon_{rb} + \eta_{2} \varepsilon_{\theta b} = 0 $$

where $\eta_{1}$ and $\eta_{2}$ are the dilatancy coefficients of these two zones, respectively.

### Elastoplastic mechanical analysis on soft-coal roadway

1. Basic equations
  According to elastoplastic mechanics, the equilibrium differential equation of the surrounding rock in various zones of the roadway can be described as:
  20 $$ \frac{{d\sigma_{ri} }}{dr} + \frac{{\sigma_{ri} }}{r} - \frac{{\sigma_{\theta i} }}{r} = 0 $$
  where the subscript “*i*” can be replaced by “*e*”, “*p*”, and “*b*” which represent the elastic zone, the plastic zone, and the broken zone, respectively.
  The geometric equations can be written as:
  21 $$ \left\{ \begin{gathered} \varepsilon_{ri} = \frac{{du_{i} }}{dr} \hfill \\ \varepsilon_{\theta i} = \frac{{u_{i} }}{r} \hfill \\ \end{gathered} \right. $$
  The physical equations are given:
  22 $$ \left\{ {\begin{array}{*{20}l} {\varepsilon_{re} = \frac{{1 - \mu^{2} }}{E}\left( {\sigma_{re} - \frac{\mu }{1 - \mu }\sigma_{\theta e} } \right)} \hfill \\ {\varepsilon_{\theta e} = \frac{{1 - \mu^{2} }}{E}\left( {\sigma_{\theta e} - \frac{\mu }{1 - \mu }\sigma_{re} } \right)} \hfill \\ \end{array} } \right. $$
  The boundary conditions for various zones of the roadway are:
  23 $$ \left\{ \begin{gathered} \sigma_{re} \left| {_{r \to \infty } } \right. = p_{0} \hfill \\ \sigma_{re} \left| {_{{r = R_{p} }} } \right. = \sigma_{rp} \left| {_{{r = R_{p} }} } \right. \hfill \\ \sigma_{rp} \left| {_{{r = R_{b} }} } \right. = \sigma_{rb} \left| {_{{r = R_{b} }} } \right. \hfill \\ \sigma_{rb} \left| {_{{r = R_{0} }} } \right. = p_{i} \hfill \\ \end{gathered} \right. $$
2. Analysis on the elastic zone
  As depicted in Fig. 12, the non-isobaric stress field of the circular roadway can be regarded as a superposition of two situations, namely a uniform compressive stress field and a stress field that is tensile on two sides and compressive on the other two sides^34,35^.

**Fig. 12:**
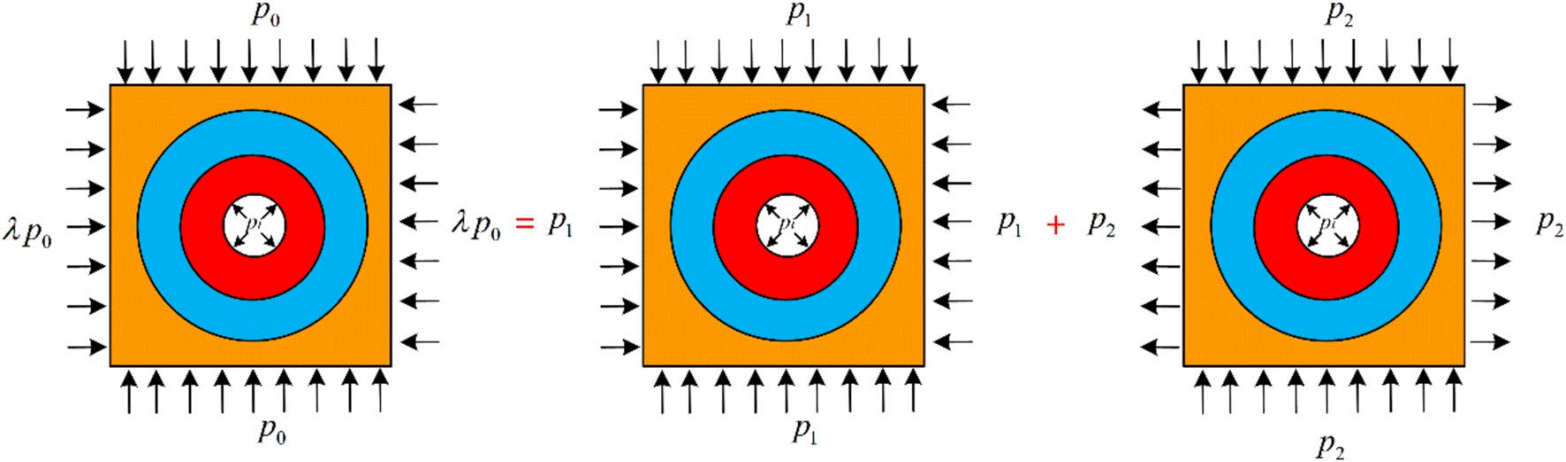
Analysis on stress fields of the surrounding rock.
  In Fig. 12,
  24 $$ p_{1} = \frac{1}{2}(1 + \lambda )p_{0} ,p_{2} = \frac{1}{2}(1 - \lambda )p_{0} $$
  By using elastic mechanics to solve the stress fields in two different situations and combining their solutions, the stress of the surrounding rock in the non-isobaric stress field can be obtained:
  25 $$ \left\{ {\begin{array}{*{20}l} {\sigma_{re} = p_{1} \left( {1 - \frac{{R_{p}^{2} }}{{r^{2} }}} \right) + \sigma_{R} \frac{{R_{p}^{2} }}{{r^{2} }} - p_{2} \cos 2\theta \left( {1 - 4\frac{{R_{p}^{2} }}{{r^{2} }} + 3\frac{{R_{p}^{4} }}{{r^{4} }}} \right)} \hfill \\ {\sigma_{\theta e} = p_{1} \left( {1 + \frac{{R_{p}^{2} }}{{r^{2} }}} \right) - \sigma_{R} \frac{{R_{p}^{2} }}{{r^{2} }} + p_{2} \cos 2\theta \left( {1 + 3\frac{{R_{p}^{4} }}{{r^{4} }}} \right)} \hfill \\ \end{array} } \right. $$
  where $\sigma_{R}$ is the radial stress at the elastic–plastic interface. When $r = R_{p}$, $\sigma_{re} = \sigma_{R}$, and the radial and tangential stresses of the surrounding rock meet the Mogi–Coulomb criterion:
  26 $$ \sigma_{R} = \frac{{2p_{1} + 4p_{2} \cos 2\theta - N_{p} }}{{M_{p} + 1}} $$
  The stress at $r = R_{p}$ is:
  27 $$ \left\{ \begin{gathered} \sigma_{re} \left| {_{{r = R_{p} }} } \right. = \frac{{(1 + \lambda )p_{0} + 2(1 - \lambda )p_{0} \cos 2\theta - N_{p} }}{{M_{p} + 1}} \hfill \\ \sigma_{\theta e} \left| {_{{r = R_{p} }} } \right. = \frac{{M_{p} (1 + \lambda )p_{0} + 2M_{p} (1 - \lambda )p_{0} \cos 2\theta + N_{p} }}{{M_{p} + 1}} \hfill \\ \end{gathered} \right. $$
  By substituting Eq. (25) into the physical equations, the strain in the elastic zone can be calculated:
  28 $$ \left\{ \begin{gathered} \varepsilon_{re} = \frac{1 + \mu }{E}\left\{ {p_{1} \left[ {(1 - 2\mu ) - \frac{{R_{p}^{2} }}{{r^{2} }}} \right] + \sigma_{R} \frac{{R_{p}^{2} }}{{r^{2} }} - p_{2} \cos 2\theta \left( {1 - 4\frac{{R_{p}^{2} }}{{r^{2} }} + 4\mu \frac{{R_{p}^{2} }}{{r^{2} }} + 3\frac{{R_{p}^{4} }}{{r^{4} }}} \right)} \right\} \hfill \\ \varepsilon_{\theta e} = \frac{1 + \mu }{E}\left\{ {p_{1} \left[ {(1 - 2\mu ) + \frac{{R_{p}^{2} }}{{r^{2} }}} \right] - \sigma_{R} \frac{{R_{p}^{2} }}{{r^{2} }} + p_{2} \cos 2\theta \left( {1 - 4\mu \frac{{R_{p}^{2} }}{{r^{2} }} + 3\frac{{R_{p}^{4} }}{{r^{4} }}} \right)} \right\} \hfill \\ \end{gathered} \right. $$
  Based on the geometric equations, the displacement of the elastic zone can be obtained:
  29 $$ u_{e} = \frac{1 + \mu }{E}\left\{ {p_{1} \left[ {(1 - 2\mu )r + \frac{{R_{p}^{2} }}{r}} \right] - \sigma_{R} \frac{{R_{p}^{2} }}{r} + p_{2} r\cos 2\theta \left( {1 - 4\mu \frac{{R_{p}^{2} }}{{r^{2} }} + 3\frac{{R_{p}^{4} }}{{r^{4} }}} \right)} \right\} $$
3. Analysis on the plastic zone.
  By combining the equilibrium equation and the Mogi–Coulomb criterion, the integral solution is calculated:
  30 $$ \sigma_{rp} = Cr^{{M_{p} - 1}} + \frac{{N_{p} }}{{1 - M_{p} }} $$
  where *C* is an undetermined coefficient. When $r = R_{p}$, $\sigma_{rp} = \sigma_{re}$, and then the expression for *C* can be obtained:
  31 $$ \left( {\sigma_{R} - \frac{{N_{p} }}{{1 - M_{p} }}} \right)R_{p}^{{1 - M_{p} }} $$
  Thus, the stress in the plastic zone can be expressed by:
  32 $$ \left\{ \begin{gathered} \sigma_{rp} = \left( {\sigma_{R} - \frac{{N_{p} }}{{1 - M_{p} }}} \right)\left( {\frac{{R_{p} }}{r}} \right)^{{1 - M_{p} }} + \frac{{N_{p} }}{{1 - M_{p} }} \hfill \\ \sigma_{\theta p} = M_{p} \left( {\sigma_{R} - \frac{{N_{p} }}{{1 - M_{p} }}} \right)\left( {\frac{{R_{p} }}{r}} \right)^{{1 - M_{p} }} + \frac{{N_{p} }}{{1 - M_{p} }} \hfill \\ \end{gathered} \right. $$
  The strain in the plastic zone consists of two parts, namely the elastic strain and the plastic strain:
  33 $$ \left\{ \begin{gathered} \varepsilon_{r} = \varepsilon_{rp} + \varepsilon_{re} \left| {_{{r = R_{p} }} } \right. \hfill \\ \varepsilon_{\theta } = \varepsilon_{\theta p} + \varepsilon_{\theta e} \left| {_{{r = R_{p} }} } \right. \hfill \\ \end{gathered} \right. $$
  By combining Eqs. (18) and (33) with the geometric equations and utilizing the continuous displacement conditions at the elastic–plastic interface, the displacement of the softening zone can be obtained:
  34 $$ u_{p} = \left[ {C_{1} - \frac{{\left( {A_{1} + \eta_{1} B_{1} } \right)R_{p} }}{{\eta_{1} + 1}}} \right]\left( {\frac{{R_{p} }}{r}} \right)^{{\eta_{1} }} + \frac{{\left( {A_{1} + \eta_{1} B_{1} } \right)r}}{{\eta_{1} + 1}} $$
  where $$A_{1} = \varepsilon_{re} \left| {_{{r = R_{p} }} = } \right.\frac{1 + \mu }{E}[p_{1} (1 - 2\mu ) - p_{1} + \sigma_{R} + 4p_{2} \cos 2\theta (\mu - 2)]$$
  $$ B_{1} = \varepsilon_{\theta e} \left| {_{{r = R_{p} }} } \right. = \frac{1 + \mu }{E}[p_{1} (1 - 2\mu ) + p_{1} - \sigma_{R} + 4p_{2} \cos 2\theta (1 - \mu )] $$
  $$ C_{1} = u_{e} \left| {_{{r = R_{p} }} } \right. = \frac{{(1 + \mu )R_{p} }}{E}\left\{ {p_{1} [(1 - 2\mu ) + 1] - \sigma_{R} + 4p_{2} \cos 2\theta (1 - \mu )} \right\} $$
  According to the geometric equations, the strain in the plastic zone is:
  35 $$ \left\{ \begin{gathered} \varepsilon_{rp} = \left[ {\frac{{\eta_{1} \left( {A_{1} + \eta_{1} B_{1} } \right)}}{{\eta_{1} + 1}}} \right]\left( {\frac{{R_{p} }}{r}} \right)^{{\eta_{1} + 1}} + \frac{{A_{1} + \eta_{1} B_{1} }}{{\eta_{1} + 1}} \hfill \\ \varepsilon_{\theta p} = \left( {\frac{{C_{1} }}{{R_{p} }} - \frac{{A_{1} + \eta_{1} B_{1} }}{{\eta_{1} + 1}}} \right)\left( {\frac{{R_{p} }}{r}} \right)^{{\eta_{1} + 1}} + \frac{{A_{1} + \eta_{1} B_{1} }}{{\eta_{1} + 1}} \hfill \\ \end{gathered} \right. $$
4. Analysis on the broken zone.
  Likewise, in the broken zone, $\sigma_{r}$ and $\sigma_{\theta }$ satisfy the equilibrium differential equation and the Mogi–Coulomb criterion, and when $r = R_{0}$, $\sigma_{rb} = p_{i}$. The stress in the broken zone is calculated as:
  36 $$ \left\{ \begin{gathered} \sigma_{rb} = \left( {p_{i} - \frac{{N_{b} }}{{1 - M_{b} }}} \right)\left( {\frac{{R_{0} }}{r}} \right)^{{1 - M_{b} }} + \frac{{N_{b} }}{{1 - M_{b} }} \hfill \\ \sigma_{\theta b} = M_{b} \left( {p_{i} - \frac{{N_{b} }}{{1 - M_{b} }}} \right)\left( {\frac{{R_{0} }}{r}} \right)^{{1 - M_{b} }} + \frac{{N_{b} }}{{1 - M_{b} }} \hfill \\ \end{gathered} \right. $$
  The displacement of the broken zone can be obtained in the same manner as the plastic zone:
  37 $$ u_{b} = \left[ {C_{2} - \frac{{\left( {A_{2} + \eta_{2} B_{2} } \right)R_{b} }}{{\eta_{2} + 1}}} \right]\left( {\frac{{R_{b} }}{r}} \right)^{{\eta_{2} }} + \frac{{\left( {A_{2} + \eta_{2} B_{2} } \right)r}}{{\eta_{2} + 1}} $$
  where $$A_{2} = \varepsilon_{rp} \left| {_{{r = R_{b} }} } \right.$$, $$B_{2} = \varepsilon_{\theta p} \left| {_{{r = R_{b} }} } \right.$$, and $$C_{2} = u_{p} \left| {_{{r = R_{b} }} } \right.$$.
5. Determination of radii of the plastic zone and the broken zone.
  In the plastic zone, Eq. (38) can be obtained on the basis of Eq. (35):
  38 $$ \varepsilon_{p} - \varepsilon_{0} = \left( {\frac{{C_{1} }}{{R_{p} }} - \frac{{A_{1} + \eta_{1} B_{1} }}{{\eta_{1} + 1}}} \right)\left( {\frac{{R_{p} }}{r}} \right)^{{\eta_{1} + 1}} + \frac{{A_{1} + \eta_{1} B_{1} }}{{\eta_{1} + 1}} - \varepsilon_{0} $$
  As mentioned earlier, in the plastic zone, $\sigma_{p}$ and $\varepsilon_{p}$ satisfy Eq. (14), then
  39 $$ \varepsilon_{p} - \varepsilon_{0} = \left[ {\frac{{2(\sigma_{p} - \sigma_{c} )(\varepsilon_{E} - \varepsilon_{0} )}}{E}} \right]^{\frac{1}{2}} $$
  When $r = R_{b}$, by substituting Eq. (38) into Eq. (39), the ratio of the radius of the broken zone to that of the plastic zone can be calculated by:
  40 $$ \frac{{R_{b} }}{{R_{p} }} = \left\{ {\frac{{C_{1} /R_{p} - (A_{1} + \eta_{1} B_{1} )/(\eta_{1} + 1)}}{{[2(\sigma_{b} - \sigma_{c} )(\varepsilon_{E} - \varepsilon_{0} )/E]^{\frac{1}{2}} - (A_{1} - \varepsilon_{0} + \eta_{1} B_{1} - \eta_{1} \varepsilon_{0} )/(\eta_{1} + 1)}}} \right\}^{{\frac{1}{{\eta_{1} + 1}}}} $$
  Additionally, when $r = R_{b}$, $\sigma_{rp} = \sigma_{rb}$. By combining Eqs. (32), (36), and (40), the calculation formulas for the radii of the plastic zone and the broken zone is obtained:
  41 $$ \left\{ \begin{gathered} R_{p} = R_{0} \left[ {\frac{{p_{i} (1 - M_{b} ) - N_{b} }}{{\sigma {}_{R}(1 - M_{b} ) - N_{b} }}} \right]^{{\frac{1}{{1 - M_{b} }}}} \hfill \\ R_{b} = R_{0} \left[ {\frac{{p_{i} (1 - M_{b} ) - N_{b} }}{{\sigma {}_{R}(1 - M_{b} ) - N_{b} }}} \right]^{{\frac{1}{{1 - M_{b} }}}} \cdot \left\{ {\frac{{C_{1} /R_{p} - (A_{1} + \eta_{1} B_{1} )/(\eta_{1} + 1)}}{{[2(\sigma_{b} - \sigma_{c} )(\varepsilon_{E} - \varepsilon_{0} )/E]^{\frac{1}{2}} - (A_{1} - \varepsilon_{0} + \eta_{1} B_{1} - \eta_{1} \varepsilon_{0} )/(\eta_{1} + 1)}}} \right\}^{{\frac{1}{{\eta_{1} + 1}}}} \hfill \\ \end{gathered} \right. $$

## Analysis on influencing factors on deformation and failure of soft-coal roadway

The practical engineering of the #842 ventilating roadway in Guobei Coal Mine in Huaibei Mining Area was investigated as a case study. The surrounding rock of this roadway was identified as soft coal, and its geological conditions were obtained: the buried depth around 850 m, the stress of original rock $p_{0}$ = 22 MPa, the lateral pressure coefficient *λ* = 1.2, the equivalent radius *R*_0_ = 2.5 m, and the support force $p_{i}$ = 0.4 MPa. According to the true triaxial loading and unloading tests, the mechanical parameters of soft coal were measured: the elastic modulus *E* = 270 MPa, Poisson’s ratio *μ* = 0.31, the internal friction angle *φ* = 38.95°, the cohesion *c* = 0.858 MPa, the plastic-zone dilatancy coefficient *η*_1_ = 1.18, and the broken-zone dilatancy coefficient *η*_2_ = 1.32. The control variable method was adopted to successively analyze the effects of the intermediate principal stress coefficient *b*, the lateral pressure coefficient *λ*, and the support force $p_{i}$ on the surface deformation of the surrounding rock, the radius of the plastic zone, and the radius of the broken zone.

### Influence of intermediate principal stress coefficient on roadway failure

**Fig. 13:**
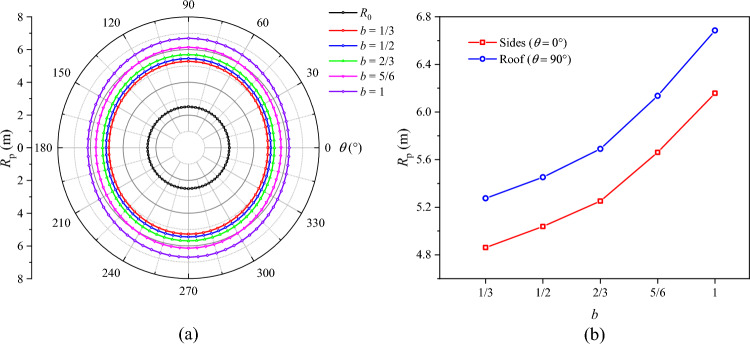
Radius of the plastic zone *R*_p_ under different intermediate principal stresses.

**Fig. 14:**
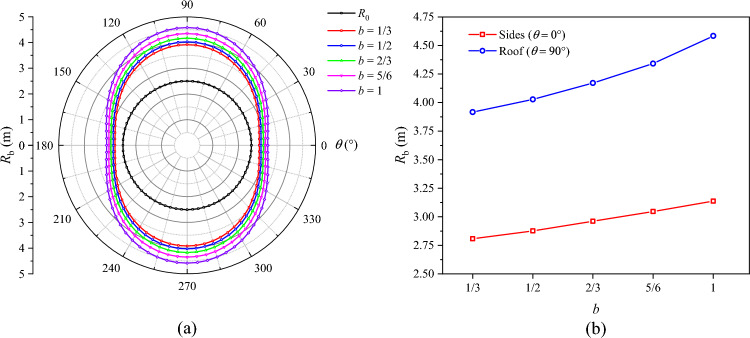
Radius of the broken zone *R*_b_ under different intermediate principal stresses.

**Fig. 15:**
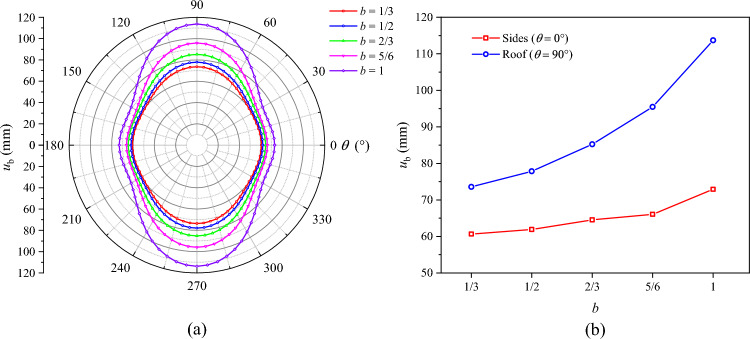
Surface displacement of the surrounding rock under different intermediate principal stresses.

To investigate the influence of the intermediate principal stress coefficient *b* on the radius of the plastic zone *R*_p_, the radius of the broken zone *R*_b_, and the surface displacement of the surrounding rock $u_{b}$, based on the true triaxial loading and unloading test scheme, the values of *b* were determined to be 1/3, 1/2, 2/3, 5/6, and 1, respectively, while the other parameters remained unchanged. With these settings, the values of *R*_p_, *R*_b_, and $u_{b}$ under different intermediate principal stress conditions were calculated and plotted (Figs. 13, 14 and 15).

In Figs. 13, 14 and 15, under different intermediate principal stress coefficients, the plastic zones are ellipse; the broken zones are elongated ellipse; and the surface displacements are distributed in the shape of a spindle. The maximum values of *R*_p_, *R*_b_, and $u_{b}$ all appear at the roof of the roadway (*θ* = 90°). As *b* increases, *R*_p_, *R*_b_, and $u_{b}$ at the roof tend to slowly increase. Specifically, when *b* = 1/3, *R*_p_, *R*_b_, and $u_{b}$ at the roof are 5.28 m, 3.92 m, and 73.6 mm, respectively. When *b* increases to 2/3, their values rise to 5.69 m, 4.17 m, and 85.3 mm, a rise of 7.76%, 6.37%, and 15.90%, respectively. When *b* > 2/3, their values start to surge. As *b* increases to 5/6, their values are 6.14 m, 4.34 m, and 95.5 mm, which are 7.90%, 4.07%, and 11.96% higher than those at *b* = 2/3, respectively. When *b* increases to 1, their values grow to 6.68 m, 4.58 m, and 113.7 mm, 8.79%, 5.53%, and 19.06% higher than those at *b* = 5/6, respectively. These results indicate that during excavation-induced unloading of surrounding rock, an increase in the intermediate principal stress will exacerbate the deformation of the surrounding rock and the dilatancy of the plastic and broken zones, exerting a noticeable impact on roadway failure.

### Influence of lateral pressure coefficient on roadway failure

**Fig. 16:**
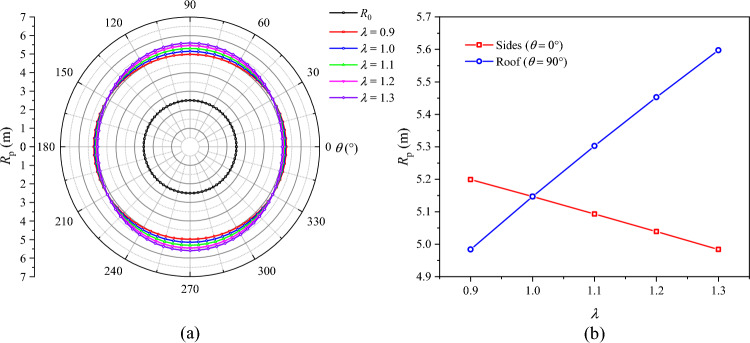
Radius of the plastic zone *R*_p_ under different lateral pressure coefficients.

**Fig. 17:**
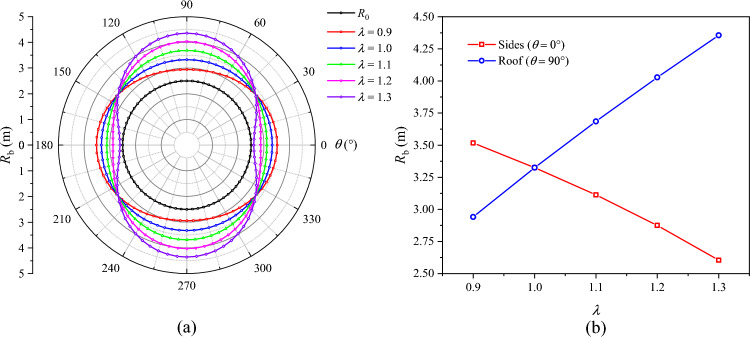
Radius of the broken zone *R*_b_ under different lateral pressure coefficients.

**Fig. 18:**
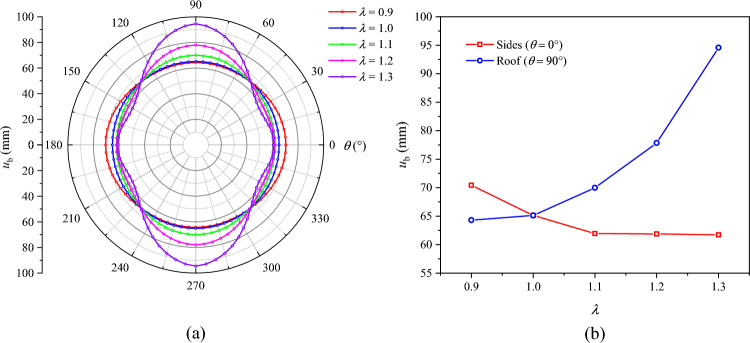
Surface displacement of the surrounding rock under different lateral pressure coefficients.

The distributions of *R*_p_, *R*_b_, and $u_{b}$ under different lateral pressure coefficients are presented in Figs. 16, 17, and 18, respectively. When *λ* = 0.9, the plastic zone, the broken zone, and the surface displacement distribution are all ellipse. The minimum values of *R*_p_, *R*_b_, and $u_{b}$ all appear at the roof of the roadway (*θ* = 90°), which are 4.98 m, 2.94 m, and 64.3 mm, respectively. Their maximum values all appear on the sides of the roadway (*θ* = 0°), which are 5.20 m, 3.52 m, and 70.4 mm, being 4.41%, 19.7%, and 9.5% higher than those at the roof, respectively. When *λ* = 1, the plastic zone, the broken zone, and the surface displacement distribution are all circular, *R*_p_, *R*_b_, and $u_{b}$ being 5.15 m, 3.32 m, and 65.1 mm, respectively. When *λ* > 1, the shapes of the broken zone and the displacement distribution begin to change with the increase in *λ*. The broken zone gradually changes from an ellipse to an elongated ellipse, while the displacement distribution gradually transitions from an ellipse to a spindle. The maximum values of *R*_p_, *R*_b_, and $u_{b}$ appear at the roof and gradually increase, while their values on the sides gradually decrease. When *λ* = 1.1, their values at the roof are 5.30 m, 3.69 m, and 70.0 mm, respectively. When *λ* = 1.3, these values grow to 5.60 m, 4.36 m, and 94.6 mm, a growth of 5.67%, 18.2%, and 35.1%, respectively. The radius of the broken zone and the surface displacement of the surrounding rock increase notably at the roof, suggesting that an increase in the lateral pressure coefficient will lead to pronounced deformation and failure of the roadway roof.

### Influence of support force on roadway failure

**Fig. 19:**
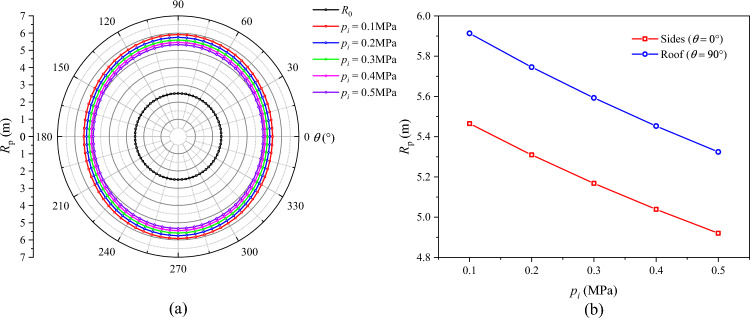
Radius of the plastic zone *R*_p_ under different support forces.

**Fig. 20:**
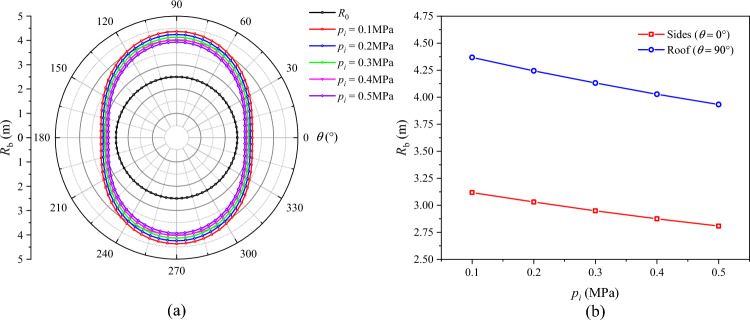
Radius of the broken zone *R*_b_ under different support forces.

**Fig. 21:**
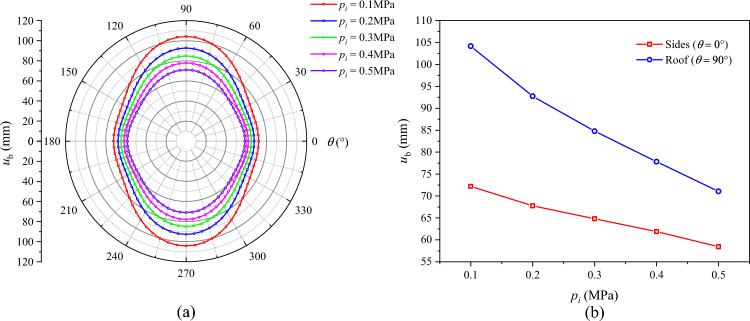
Surface displacement of the surrounding rock under different support forces.

The distributions of *R*_p_, *R*_b_, and $u_{b}$ under different support force conditions are displayed in Figs. 19, 20, and 21, respectively. On the whole, the plastic zone, the broken zone, and the surface displacement distribution are in the shape of an ellipse, an elongated ellipse, and a spindle, respectively. The maximum values of *R*_p_, *R*_b_, and $u_{b}$ all appear at the roof, and the minimum values all appear on the sides. As the support force *p*_*i*_ increases, the plastic zone, the broken zone, and the surface displacement of the roadway gradually shrink. When *p*_*i*_ = 0.1 MPa, the *R*_p_, *R*_b_, and $u_{b}$ at the roof are 5.91 m, 4.37 m, and 104.2 mm, respectively. As *p*_*i*_ increases to 0.3 MPa, their values at the roof drop to 5.59 m, 4.13 m, and 84.8 mm, by 5.41%, 5.49%, and 18.6%, respectively. When *p*_*i*_ increases to 0.5 MPa, these values decline to 5.32 m, 3.93 m, and 71.1 mm, a further decline of 4.83%, 4.85%, and 16.2%, respectively. Hence, it can be concluded that during the excavation of a soft-coal roadway, the deformation and failure of its surrounding rock can be controlled by increasing the support force.

## Conclusions

The deformation and strength characteristics of soft coal under true triaxial loading and unloading conditions were investigated, and the constitutive relationship and strength criterion of soft coal under true stress paths in roadway excavation were determined. On this basis, analytical solutions for the displacement of the surrounding rock, the radius of the broken zone, and the radius of the plastic zone were derived. Finally, in accordance with a practical engineering case, the effects of different influencing factors on the deformation and failure characteristics of a soft-coal roadway were analyzed. The following main conclusions were drawn.

1. The stress–strain curves of soft coal in the true triaxial loading and unloading tests can be divided into four stages, namely the elastic stage, the pre-peak plasticity-strengthening (hardening) stage, the post-peak plasticity-weakening (softening) stage, and the instability failure stage. The stress–strain relationship in the pre-peak hardening and post-peak softening stages follows a quadratic function. As the initial $\sigma_{2}$ increases, the difference between strains in the two unloading directions gradually enlarges, making the coal more prone to dilatancy and deformation in the $\sigma_{3}$ direction and thereby decreasing its peak strength.
2. Compared to the Mohr–Coulomb criterion and the Drucker–Prager criterion, the Mogi–Coulomb criterion can more accurately describe the strength characteristics of soft coal under true triaxial loading and unloading paths.
3. Analytical solutions for the displacement of the surrounding rock, the radius of the broken zone, and the radius of the plastic zone in soft-coal roadways under excavation stress paths were derived after taking the nonlinear strain strengthening and softening characteristics of soft coal, the Mogi–Coulomb criterion, the intermediate principal stress, and the surrounding rock dilatancy characteristics into account.
4. An increase in the intermediate principal stress coefficient *b* will aggravate the deformation of the surrounding rock and the dilatancy of the plastic and broken zones. Meanwhile, an increase in the lateral pressure coefficient *λ* can bring about a gradual increase in the deformation degree of the plastic and broken zones at the roof and a decrease on the sides. The shape of the broken zone and surface displacement distribution will change correspondingly. Moreover, as the support force *p*_*i*_ increases, the plastic zone, broken zone, and the surface displacement of the roadway all gradually shrink.

## Data Availability

The data used to support the findings and results of this study are available from the corresponding author upon request.
